# Case Report: Traumatic anterior cerebral artery aneurysm in a 4-year old child

**DOI:** 10.12688/f1000research.7028.1

**Published:** 2015-09-15

**Authors:** Sunil Munakomi, Karuna Tamrakar, Pramod Chaudhary, Binod Bhattarai, Iype Cherian

**Affiliations:** 1 College of Medical Sciences, Chitwan, Nepal

**Keywords:** trauma, pseudo-aneurysm, cerebral angiography

## Abstract

Traumatic intracranial aneurysm in the proximal part of the anterior cerebral artery in the pediatric population has not been documented so far. Here we report the case of a 4 year-old child who developed a pseudo-aneurysm after minor head trauma and was managed successfully with trapping of the aneurysm. A ventriculo-peritoneal shunt was placed as the child became dependent on extraventricular drain during the post-operative period. The patient made excellent recovery in neurological status within 1 month of post-operative clinical follow up.

## Introduction

Overall incidence of traumatic intracranial aneurysm (TICA) is 1% and is usually associated with penetrating head injury or contagious skull fracture ^1^. The first reported pediatric TICA was in 1829; a right middle meningeal artery aneurysm in an autopsy report of 12 year-old boy with blunt head injury on the right temporal region ^2^. Prevalence of TICA is more among males, with a ratio of 2:1 or 3:1 relative to females, most likely reflecting the greater frequency of trauma among males. The majority of cases are associated with trauma, with approximately 30% of reported cases occurring in children and adolescents before the age of 20. Petrous or cavernous part of internal carotid artery aneurysm (ICA) is associated with skull base fracture. Supraclinoid aneurysm may develop due to blunt arterial contusion by the anterior clinoid process or sudden stretching of the artery during impact to the head. Posterior circulation TICA can develop either due to direct osseous injury or stretching or compression of an artery against the tentorium. However, non-traumatic aneurysms are rare in the pediatric population; the relative frequency of trauma-induced aneurysms in children is high. Most common locations are distal vasculatures like anterior cerebral artery (ACA) (38%), petrous, cavernous, supraclinoid ICA (29%), distal branch of middle cerebral artery (MCA) (25%), and vertebrobasilar system (8%) ^3^. Here we report the case of a 4 year-old girl who presented with delayed intracranial hemorrhage from a ruptured traumatic aneurysm involving the proximal anterior cerebral artery, managed by trapping of the aneurysm.

## Case presentation

**Figure 1.**
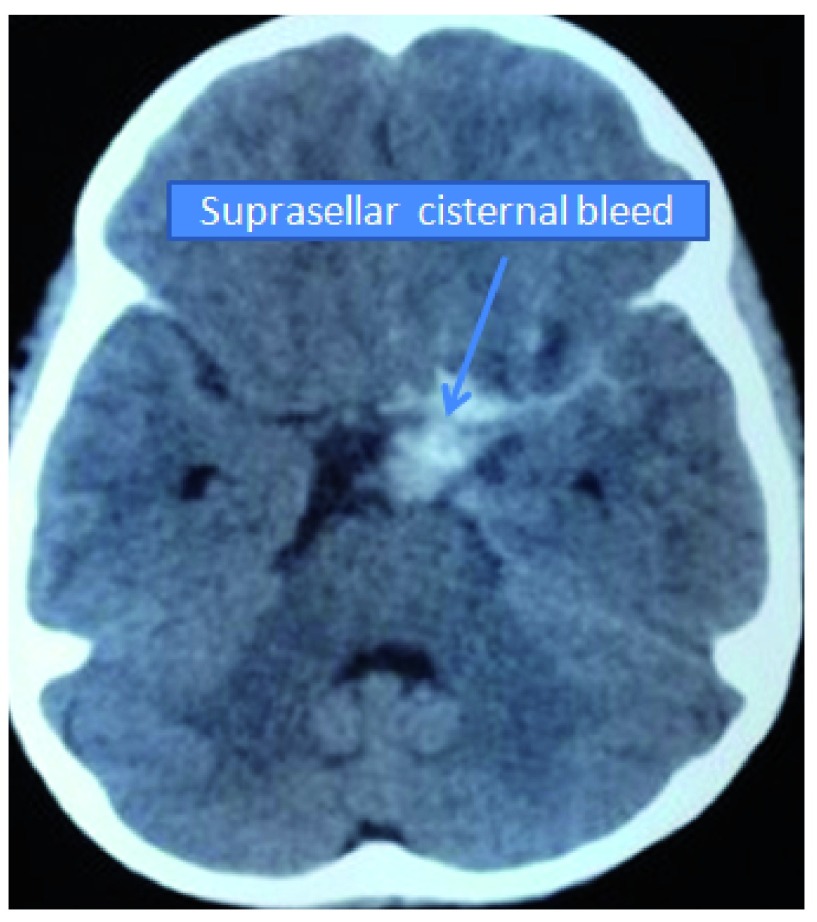
Initial CT scan showing the region of the bleed.

**Figure 2.**
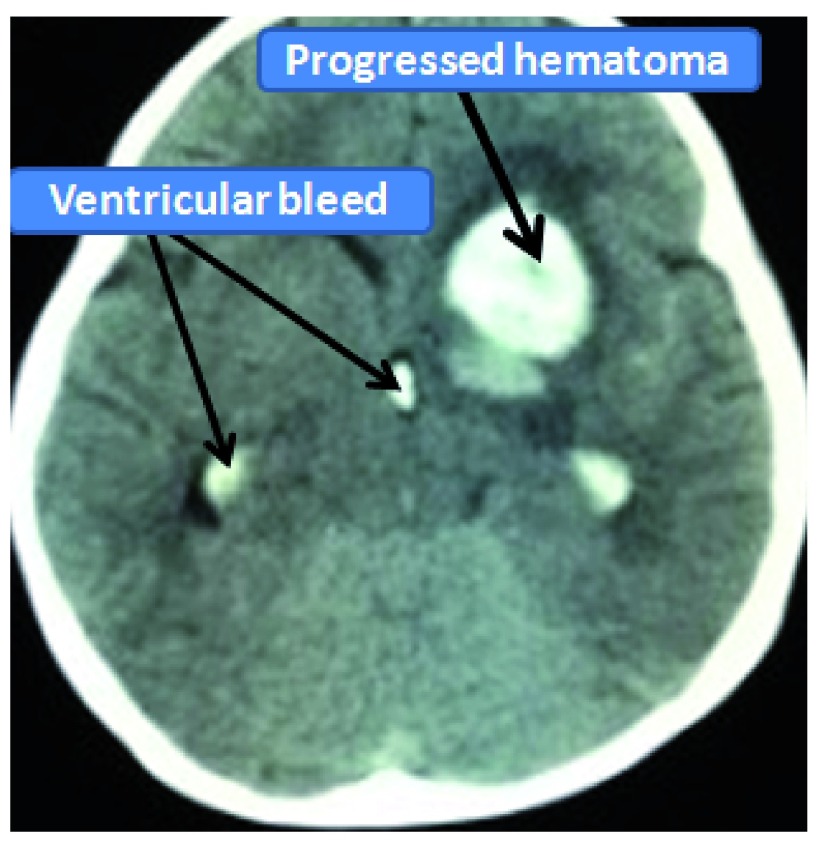
CT head showing evidence of re-bleed in the previous territory along with ventricular extension.

**Figure 3.**
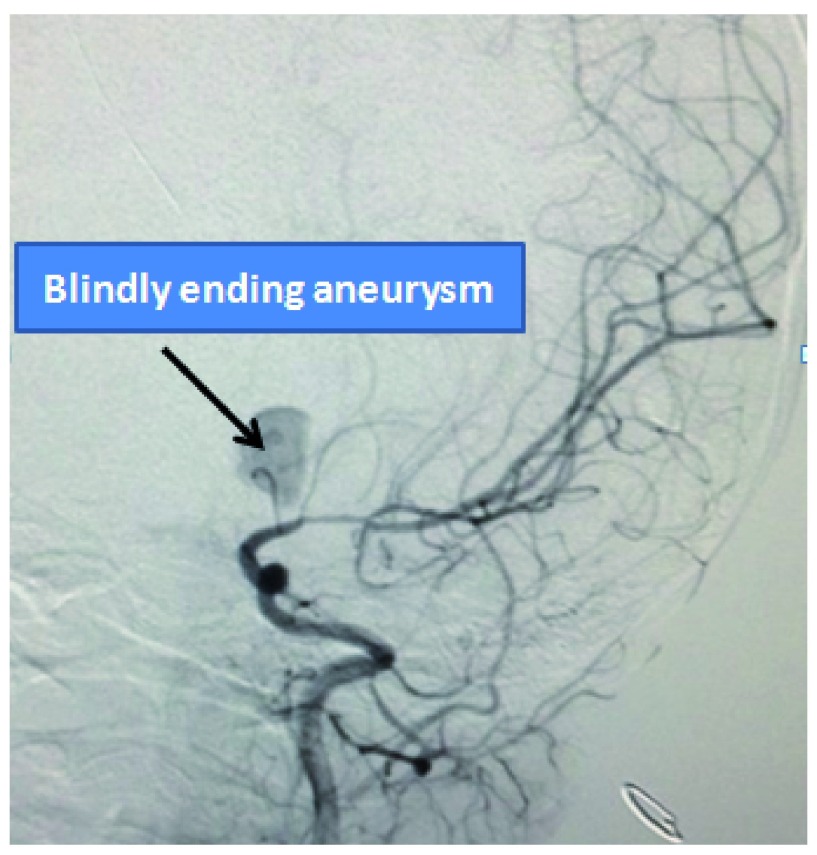
Diagnostic angiography showing the presence of blind left A1 aneurysm.

**Figure 4.**
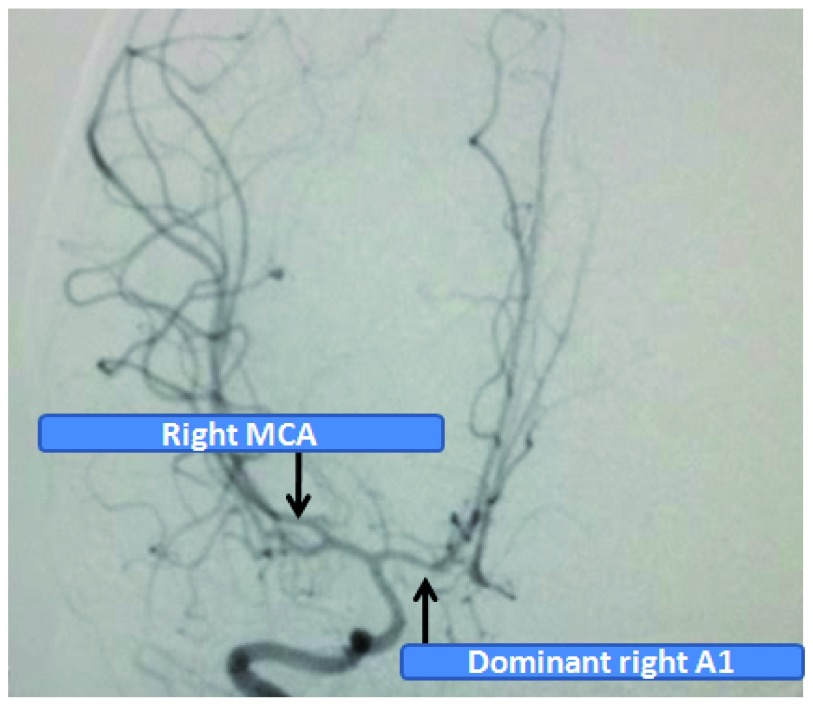
Angiography revealing both A2’s supplied by the right A1.

**Figure 5.**
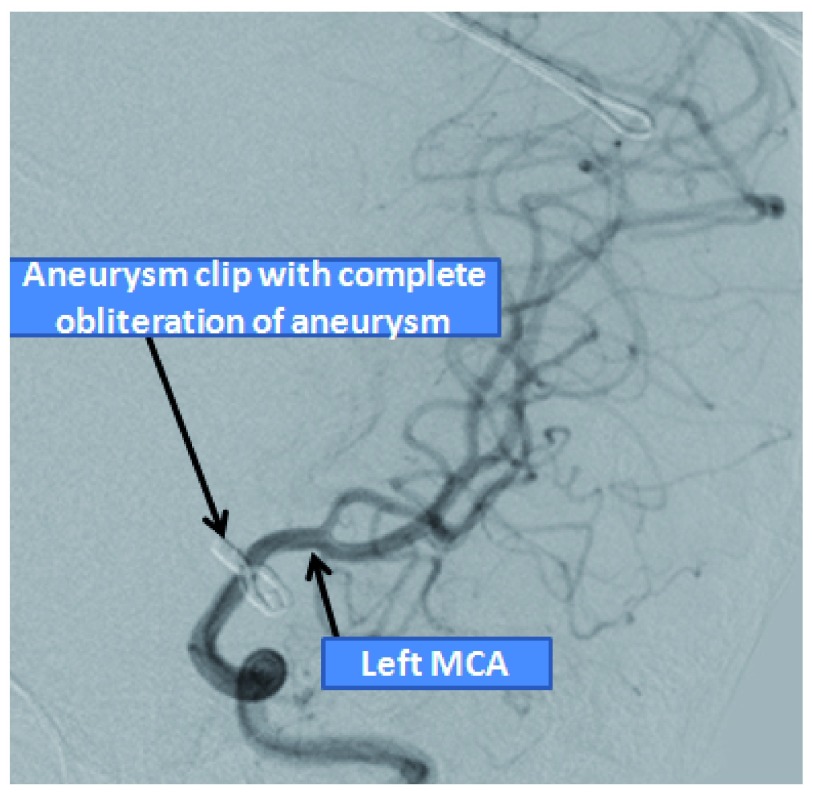
Post-operative angiography showing complete obliteration of the blind aneurysm.

**Figure 6.**
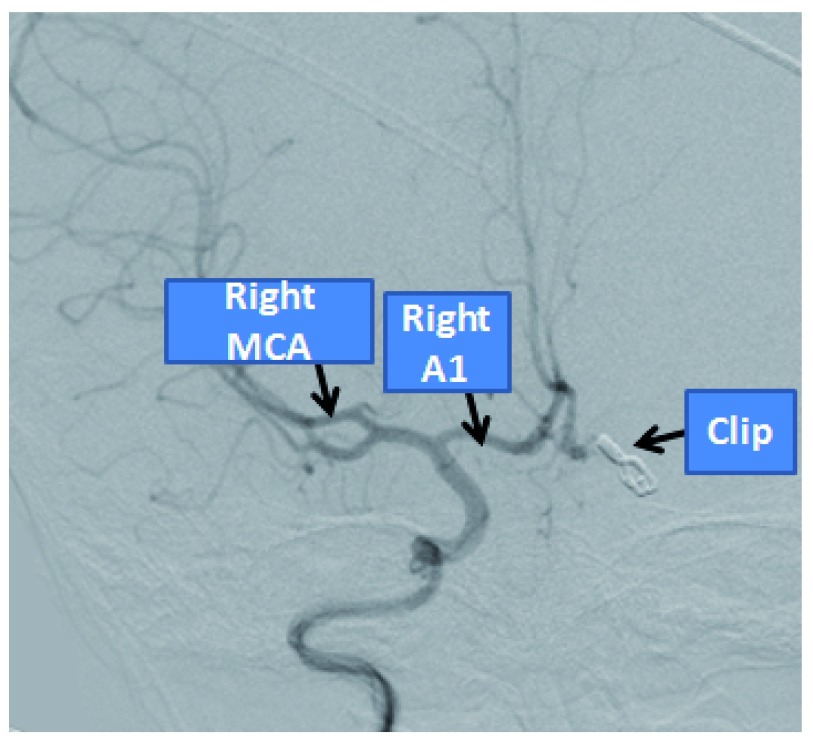
Angiography showing both A2’s supplied from the right A1.

A 4 year-old girl from Sarlahi district in Nepal was brought to our emergency room with a history of sudden onset of severe headache and generalized tonic clonic seizures. She had a history of a minor fall injury while playing at preschool. At that time there was no loss of consciousness, nausea or vomiting and she remained well for the following two days. Two days later at around 3 a.m., the child screamed out in her sleep and complained of severe headache followed by an episode of generalized tonic clonic seizure. The child was then rushed to the hospital. At presentation, her Glasgow Coma Score was 14/15 (E4/M6/V5). No anisocoria was present. She was hemiparetic on the right side with a power grade of 3/5. Computed tomography (CT) scan of the head showed focal intra cerebral hemorrhage in the medial basifrontal region and subarachnoid hemorrhage (SAH) in the inter-hemispheric and in the left sylvian fissure ( Figure 1). No significant past medical or surgical illnesses were elicited. She was managed conservatively in the neurosurgical intensive care unit. In repeated serial CT scans, the hematoma was found to be resolving and the child’s motor power in the right side had improved to 4+/5. The child was discharged with advice of regular follow up. The child was again brought to the emergency room one month following the initial hemorrhage with a history of headache and repeated episodes of vomiting. GCS was 15/15 with both pupils equal and reacting to light. CT scan showed re-bleed in the left medial basifrontal region with ventricular extension (Graeb score of 8/12) ( Figure 2). The child developed acute hydrocephalus with sudden drop in conscious level which was managed with emergent placement of external ventricular drain (EVD) from the right Kocher’s point. Conventional cerebral angiography showed delayed filling of a 9.6 mm × 6.8 mm aneurysm arising from the proximal part of the left anterior cerebral artery without a discrete neck ( Figure 3). Left ACA complex was not visualized except the aneurysmal sac. Both the distal anterior cerebral complex i.e. A2 segments were filled via the right anterior cerebral artery ( Figure 4). Left pterional craniotomy and trapping of the aneurysm was performed without any intraoperative complications. H complex was redefined and A1 was found to be blind which itself was a culprit for repeated rupture. Post-operatively the child remained irritable whenever the EVD drainage was clamped off and repeated CT scans revealed persistent hydrocephalus. She was therefore managed with a right ventriculo-peritoneal shunt. On the day of discharge, 23 days after admission, she was playful with grade 4+ power of right sided limbs which became normal in the 2 week follow up period. The child made excellent recovery during 1 month of clinical follow up with no focal neurological deficit and remained asymptomatic. Angiographic follow up after 3 months showed complete obliteration of the aneurysm ( Figure 5; Figure 6).

## Discussion

TICA in pediatric populations is rare and accounts for 0.5–4.6% of all aneurysms ^4^. These aneurysms are more vulnerable to bleed than true aneurysms due to the lack of a true wall and also the neck, so that they usually present with intracranial hemorrhage (ICH). ICH has been reported in > 60% of TICA cases ^5^. Associated mortality among TICA patient in the pediatric age group has been reported as high as 50% ^6^. TICA is usually located in the periphery and appears to be irregular in shape without visualization of a defined neck. The most common location of TICA is the distal anterior cerebral artery and middle cerebral artery. Petrous or cavernous segment of ICA is also frequently involved. Anterior circulation is more involved than posterior circulation which accounts for less than 10% of all TICA ^7^. Due to the lack of an endothelial layer in the aneurysm, they more likely result from penetrating injuries rather than closed head injuries. Non penetrating injuries are usually secondary to acute shearing forces as in rapid deceleration injury or due to skull fracture with underlying dural or cortical contusions. They usually develop as a consequence of sudden stretching or compression against the rigid dural structure such as the falx or the tentorium. Minor head injury without significant brain parenchymal injury or bony fractures may also cause vascular injuries which later develop into a pseudo-aneurysm.

In PubMed ( www.pubmed.gov), a search of “pediatric traumatic intracranial aneurysm” returned 27 cases, and among them 10 were of patients aged 0–4 ^7– 11^. 7 were of the age 5–10 ^1, 3, 5^ and 8 were 11–18 years old ^1, 10, 12^. 12 were males and 7 were females whereas the remaining cases were mentioned as only infant or child ^8, 9, 11^. 21 children presented with history of traumatic brain injury; 2 had sustained gunshot injury ^13, 14^, 2 cases presented with TICA following VP shunting and craniopharyngioma surgery ^15, 16^. An additional 2 cases developed aneurysm due to shaken baby syndrome ^10, 17^. The most frequently involved vessel was the distal anterior cerebral artery (11 cases) ^7^, followed by the petrous segment internal carotid artery (5 cases) ^9^, distal middle cerebral artery (2 cases) ^3^, posterior cerebral artery (2 cases) ^5^, posterior inferior cerebellar artery (1 case) ^18^, basilar artery (2 cases), vertebral artery (2 cases) ^1, 19^, common carotid artery (1 case) ^19^ and superficial temporal artery (1 case) ^20^. Involvement of posterior circulation was relatively uncommon as compared with anterior circulation vessels. Traumatic aneurysm of proximal ACA in pediatric populations has not been documented in English literature so far. This is the first reported case of TICA of proximal ACA in a child under 5 years of age. Congenital aneurysms are true aneurysms that occur in the branching site of the circle of Willis. They are very rare in the pediatric population as compared to traumatic dissection. Based on the clinical-radiological aspect, the author’s reported case should be the traumatic dissection of ACA leading to pseudo-aneurysm rather than that of congenital origin.

Management has mostly been confined to surgical clipping, and few reports involve the endovascular treatment of aneurysm. Among 18 treated cases; 8 cases underwent clipping ^3, 7, 20, 21^, trapping was done in 4 cases ^10, 21^ and coil embolization was performed in 6 cases ^16, 21^. Parent vessel occlusion was done in 3 cases ^21^. Spontaneous thrombosis had occurred in 2 cases ^1, 5^ and 2 children died regardless of treatment ^22^. Although spontaneous complete occlusion of TICA is thought to be extremely rare, 15% of spontaneous thrombosis of TICA has been reported in the literature ^23^. Pseudo-aneurysms generally do not have discrete necks and are often friable so that surgical clipping may not be an option. Trapping, in which clips are placed on the parent vessel both proximal and distal to the aneurysm, followed by aneurysmal excision, is a preferred method of treatment. Clinical outcome in a pediatric patient with TICA depends on severity of the injury, with a potentially high mortality from rupture or re-bleeding of the aneurysm ^22^.

## Conclusion

TICA occurrence in the pediatric population is very low. Delayed presentation of intracranial hemorrhage with acute deterioration after minor head trauma in the pediatric age group warrants cerebral angiography for proper diagnosis and management. Trapping of traumatic aneurysm arising from the main arterial trunk is a more tenable procedure. Arterial reconstruction or bypass may prove to be acquiescent in some complex circumstances.

## Consent

Both written and verbal informed consent for publication of images and clinical data related to this case was sought and obtained from the mother of the patient.
