# Supplementary material for: Case Report: Traumatic anterior cerebral artery aneurysm in a 4-year old child
Source: F1000Res. 2015 Sep 15;4:804. [Version 1] doi: 10.12688/f1000research.7028.1 (PMC5017288; doi:10.12688/f1000research.7028.1)
Supplement: Peer review history

# Peer review: Referee response for version 1

**DOI:** 10.5256/f1000research.7566.r16091

**Published:** 2016-09-07

**Authors:** Guru Dutta Satyarthee

**Affiliations:** 1 Department of Neurosurgery, All India Institute of Medical Sciences, New Delhi, India

Authors report an extremely uncommon entity of postraumatic aneurysm in a child, who developed hydrocephalus and showed rapid neurological worsening, managed with the placement of an external ventricular drain, followed by successful surgical management of surgery of aneurysm. The index case, a four-year-old girl, presented with a history of sudden onset severe headache in association with generalized tonic clonic seizures with antecedent history of a minor fall. On examination, the Glasgow coma score was 14, right sided  hemiparesis, a CT scan of the head revealed presence of subarachnoid hemorrhage in the inter-hemispheric fissure and left sylvian fissure and associated intracerebral hemorrhage in the basifrontal region, however, a vascular aetiology was not suspected and managed conservatively. Ideally digital substraction angiography ( DSA) should have been carried out and managed accordingly to the findings. She again presented after one month with re-bleed, required external ventricular drain placement for hydrocephalus. DSA revealed an aneurysm in the region of left anterior cerebral artery, although both distal anterior cerebral arteries were filling with right internal carotid artery injection, and underwent craniotomy and trapping of the aneurysm.  

 Hydrocephalus development is a common and devastating complication of aneurysmal subarachnoid hemorrhage with incidence of 20% to 30% cases. Onset of hydrocephalus can be acute as early as within 48 hours or delayed by up to weeks following ictus. Early recognition of symptoms and signs and computed tomography findings are important for planning of management. As cases showing rapid deterioration need  external ventricular drain, to tide the crisis, which may be converted to ventriculo-peritoneal shunt as it aids in management of raised intracranial pressure, helps in clearance of intraventricular blood. ^1^^,^^2^

 Intracranial traumatic aneurysm is a relatively rare clinical entity caused by blunt, penetrating, or iatrogenic head trauma ^3^^,^^4^. It can be classified histologically as false or true aneurysm. In false aneurysms or  pseudoaneurysm disruption involves all layers of the arterial wall associated with the presence of hematoma in the surrounding soft tissue that prevents extravasations of blood and is considered the most common form of traumatic intracranial aneurysms ^5^. In true aneurysms, the intimal layer, internal elastic lamina and media layers are disrupted but with an intact tunica adventitia. The intracranial space into which the hemorrhage occurs depends on the vessel and the segment of the vessel involved. Once an extracranial artery becomes intradural, the external elastic lamina disappears transforming the arterial wall into a relative weak vessel. ^4^^,^^5^ 

 The reported risk of hemorrhage in posttraumatic pseudoaneurysm is 19%, with a peak incidence of rupture at 2–3 weeks after the injury, resulting in 32–54% mortality. ^5^

 Treatment options include surgical repair and endovascular interventions. The treatment approach is often needed to be a tailor made approach for individual cases, and depends on factors such as atypical locations, irregular shape, relation with parent vessel and branches, age of patient, and shapes are a factor ^1^^,^^3^^,^^6^. Miley *et al. *analysed DSA of 74 patients with the diagnosis of closed head injury, vascular injury detected in 24 cases and out of which four cases had total of 4 traumatic intracranial pseudoaneurysm, were located in the supraclinoid segment of the internal carotid artery in two case, one in the cavernous segment   and rest one in the paraophthalmic segment ^6^.

I have read this submission. I believe that I have an appropriate level of expertise to confirm that it is of an acceptable scientific standard.

## References

[1] GermanwalaAVHuangJTamargoRJ: Hydrocephalus after aneurysmal subarachnoid hemorrhage. *Neurosurg Clin N Am*.2010;21(2) : 10.1016/j.nec.2009.10.013 263-70 10.1016/j.nec.2009.10.013 20380968

[2] SatyartheeGDMahapatraAK: Is subduro-peritoneal shunt surgery the first or last resort in managing subdural effusion developing after supratentorial tumor surgery in infancy?. *J Pediatr Neurosci*.10(1) : 10.4103/1817-1745.154371 83-5 10.4103/1817-1745.154371 25878758PMC4395960

[3] LiuPYangMCaiMQinJPanL: Treatment of Pediatric Traumatic Intracranial Pseudoaneurysm Using Endovascular Covered Stent: Three Case Reports. *World Neurosurg*.2016;88: 10.1016/j.wneu.2015.12.037 693.e1-6 10.1016/j.wneu.2015.12.037 26724623

[4] WilkinsonIM: The vertebral artery. Extracranial and intracranial structure. *Arch Neurol*.1972;27(5) :392-6 507889510.1001/archneur.1972.00490170024004

[5] AsariSNakamuraSYamadaOBeckHSugataniH: Traumatic aneurysm of peripheral cerebral arteries. Report of two cases. *J Neurosurg*.1977;46(6) : 10.3171/jns.1977.46.6.0795 795-803 10.3171/jns.1977.46.6.0795 859016

[6] MileyJTRodriguezGJQureshiAI: Traumatic Intracranial Aneurysm Formation following Closed Head Injury. *J Vasc Interv Neurol*.2008;1(3) :79-82 22518228PMC3317295

# Peer review: Referee response for version 1

**DOI:** 10.5256/f1000research.7566.r15779

**Published:** 2016-08-30

**Authors:** Venkatesh S. Madhugiri

**Affiliations:** 1 Division of Neurosurgery, Tata Memorial Centre, Mumbai, India

This is an interesting case report of a complex aneurysm. However, a few comments on some aspects of the paper are presented here

1. Trivial trauma of the kind described does not usually lead to aneurysmal rupture. Could the rupture of the aneurysm have caused the fall? Moreover, when the CT after the first ictus (the trivial fall) disclosed thick subarachnoid hemorrhage in the Sylvian and suprasellar cistern rather over the convexity of the hemispheres (as would usually be expected with trauma), did the authors not consider doing a DSA or CTA in the first instance?
2. It is very unusual for a major vessel to blindly end in an aneurysm. There is generally a distal take-off of the distal vessel even from giant aneurysms. This is borne out by the fact that the L A2 was being filled across the ACoA on the R ICA injection. Have the authors considered the possibility that this was an A1 aneurysm? The proximal A1 could have been partly thrombosed due to extension of thrombus from the aneurysm into the parent vessel. This could explain the poor visualization of the L A1 whereas the L A2 distal to the aneurysm was being filled by the ACoA.
3. The authors should describe the intra-operative findings in slightly greater detail. The L A1 was blind, per the authors. Was there no connection between the L A1 and A2? It is more likely that the aneurysm obscured this vessel. Was the aneurysm thrombosed? Did it appear to be a true aneurysm or pseudo-aneurysm? Was the sac opened after clipping and dissected away from surrounding structures to clarify the anatomy?
4. The preop DSAs shows vasospasm of the L M1. The authors should comment on what treatment, if any, was considered or given for this. The spasm appears to have resolved on the postop DSA. When was this obtained?
5. Pediatric traumatic intracranial aneurysms are indeed rare. In the Discussion the authors state "a search of “pediatric traumatic intracranial aneurysm” returned 27 cases." However, PubMed search strategies need to built more carefully if valid results are to be obtained. A cursory search using the strings  "((aneurysm) AND intracranial) AND trauma" and subsequently applying a filter for only children (birth-18 years) returned 584 results. Even if only 10% of these results were relevant to the search, this would still result in 58 papers dealing with this issue.

The full search employed was - 

 (("aneurysm"[MeSH Terms] OR "aneurysm"[All Fields]) AND intracranial[All Fields]) AND ("injuries"[Subheading] OR "injuries"[All Fields] OR "trauma"[All Fields] OR "wounds and injuries"[MeSH Terms] OR ("wounds"[All Fields] AND "injuries"[All Fields]) OR "wounds and injuries"[All Fields]) AND ("infant"[MeSH Terms] OR "child"[MeSH Terms] OR "adolescent"[MeSH Terms])

I have read this submission. I believe that I have an appropriate level of expertise to confirm that it is of an acceptable scientific standard, however I have significant reservations, as outlined above.

# Peer review: Referee response for version 1

**DOI:** 10.5256/f1000research.7566.r10317

**Published:** 2015-09-22

**Authors:** Naoya Kuwayama

**Affiliations:** 1 Department of Neurosurgery, Graduate School of Medicine and Pharmacological Science, University of Toyama, Toyama, Japan

This is a case report of a 4-year-old child with a delayed intracerebral hematoma resulting from the traumatic pseudoaneurysm of the left A1 segment.

As authors emphasized, this is a very rare case occurring in childhood and the management of the patient, including the surgical treatment, was excellent. In the discussion, the epidemiology, diagnosis, and treatment option of the traumatic intracranial aneurysms were briefly but well documented.

Finally, this article can be considered as acceptable, although the following points should be addressed:

1. They should discuss the possibility that the pseudoaneurysm had already developed when the child first complained of a headache 2 days after head injury.
2. They should describe the details about the surgical method focusing how they trapped the aneurysm with one clip. Did they clip only the neck or A1 segment itself?
3. I cannot understand what “H complex was redefined” means in the operation.

I have read this submission. I believe that I have an appropriate level of expertise to confirm that it is of an acceptable scientific standard.
